# Family Therapy for Anorexia Nervosa With Emerging Adults: A Retrospective Case Series in Routine Clinical Care

**DOI:** 10.1002/erv.70107

**Published:** 2026-05-15

**Authors:** D. Nursigadoo, E. Dodge, K. L. Allen, U. Schmidt, J. Baudinet

**Affiliations:** ^1^ Adult Eating Disorders Service South London and Maudsley NHS Foundation Trust London UK; ^2^ Centre for Research in Eating and Weight Disorders (CREW) Institute of Psychiatry, Psychology and Neuroscience (IoPPN) King's College London London UK; ^3^ Maudsley Centre for Child and Adolescent Eating Disorders (MCCAED) South London and Maudsley NHS Foundation Trust London UK

**Keywords:** anorexia nervosa, emerging adult, family therapy for anorexia nervosa, family‐based treatment, transition, young adult

## Abstract

**Aim:**

To broaden treatment options for emerging adults with anorexia nervosa we explored the applicability of an adapted Family Therapy for Anorexia Nervosa (FT‐AN‐EA) in a routinely collected outpatient case series.

**Method:**

Thirty emerging adults (aged 18–25) diagnosed with anorexia nervosa or atypical anorexia nervosa were recruited and agreed to participate in FT‐AN‐EA. Participant demographics, treatment characteristics (treatment duration, number of sessions, treatment non‐completion, need for inpatient care) and body mass index (BMI) at assessment and discharge were collected.

**Results:**

The series comprised of 21 new referrals to the adult eating disorder service and 9 age‐related transitions from child and adolescent services (CAEDS). Two patients were classified as early non‐completers (attending four sessions or less of FT‐AN‐EA). In the treatment‐completers group (*n* = 28), mean duration of treatment was 10 months (22 sessions). 20 completers (71.4%) restored weight to BMI > 18.5 kg/m^2^ at discharge. 24 completers (85.7%) were discharged to no further care (general practitioner [GP] only), two (7.1%) were referred onto other interventions and two (7.1%) were admitted to inpatient care.

**Conclusion:**

This case series provides preliminary evidence for the applicability of adapted FT‐AN for emerging adults with anorexia nervosa. Further studies, under more controlled conditions, are needed to establish its efficacy and identify optimal treatment adaptations for this population.

## Highlights

- Family therapy for anorexia nervosa adapted to the needs of emerging adults (FT‐AN‐EA) is a potentially useful intervention for this population.
- Treatment length is similar to FT‐AN for children and adolescents.
- Most emerging adults receiving FT‐AN‐EA did not need further eating disorder treatment.

## 1 Introduction and Aims

Emerging adulthood (age 18–25) is a developmentally sensitive time. At this stage, young people are legally seen as independent adults, but often still rely heavily on emotional, practical and financial support from their families, especially if they have an eating disorder (Potterton et al. 2020, 2025). Around half of newly arising cases of anorexia nervosa (AN) have their onset in emerging adulthood (Solmi et al. 2022). Studies have not yet examined the most effective format of treatment for this age group, with the majority of research being focused on individual treatments.

Whilst eating disorder focused family therapy is recommended internationally as the first line treatment for AN and atypical anorexia nervosa (A‐AN) for young people below age 18 (Crone et al. 2023; NICE 2017), individual therapies are recommended for adults, including emerging adults (NICE 2017), with adjunctive support for families and significant others (Hannah et al. 2022; Langley et al. 2018). This age‐based division of treatment and care has recently been challenged with the suggestion that it should be replaced with a more flexible, developmentally informed approach (U. H. Schmidt et al. 2025; Wade 2023). In line with this, the American Psychiatric Association (APA) Guidelines have recommended that, where emerging adults have an involved care giver they should: …‘be treated with ED‐focused family based treatment, which should include caregiver education aimed at normalising eating and weight control behaviours and restoring weight’ (Crone et al. 2023, 168).

As yet, consensus is lacking as to what constitutes the effective developmentally appropriate inclusion of family members in the treatment of emerging adults with eating disorders. It is common to include some parent/carer support alongside individual adult approaches, however, it is unclear whether a conjoint family approach is as effective as an individual approach (Fleming et al. 2021). For example, the First Episode Rapid Intervention for Eating Disorders (FREED) service model and care pathway for recent‐onset eating disorders in emerging adults places a strong emphasis on family involvement while delivering individual, evidence‐based therapies (Allen et al. 2020). Within the Maudsley Model of AN Treatment for Adults (MANTRA; U. Schmidt et al. 2023), one of the NICE (2017) recommended first line treatments for adults, including parents/carers in the collaborative development of the formulation and in at least two sessions during treatment is strongly encouraged (U. Schmidt et al. 2015).

Despite this increasing inclusion of parents/carers in an adjunctive role during treatment, there is a limited evidence base for the efficacy of a conjoint family approach for emerging adults (Dodge et al. 2024). Three studies have reported positive outcomes (Chen et al. 2016; Dimitropoulos et al. 2018; Nyman‐Carlsson et al. 2020). Two open studies (*n* = 22 and 26, respectively) adapted family‐based therapy (FBT) (Lock and Le Grange 2012) for young adults (age 18 to 25/26, respectively) (Chen et al. 2016; Dimitropoulos et al. 2018). Adaptations made for this age group were the inclusion of friends/roommates rather than only parents, the importance of a flexible, collaborative therapeutic stance, a focus on developmental issues, a need to address the discomfort with and temporary nature of increased parental support and the offer of individual sessions in the latter phases of treatment. Both studies reported improvements in weight, comparable to that in adolescent studies, as well as improvements in eating disorder psychopathology, broader psychological functioning. However, in Chen et al.'s (2016) study drop‐out rate was high (9/22, 40.9%) and treatment duration was shorter‐than‐expected.

Additionally, one randomised controlled trial (RCT, *N* = 78; Nyman‐Carlsson et al. 2020) found family therapy for young adults, manualised and adapted from FBT (Lock and Le Grange 2012), to be as effective as individual cognitive behavioural therapy (CBT) with excellent recovery rates in both groups. Although no formal cost‐effectiveness analysis was conducted, CBT was less costly, requiring only about a third of the therapist time. Differences in the model used in this study from the adolescent FBT model included, a longer treatment duration (18 vs. 12 months), session length (90 vs. 60 minutes), a higher number of individual sessions offered, two therapists per session, and no family meal session.

The primary aim of this brief report is to evaluate the applicability of adapted Maudsley Family Therapy for Anorexia Nervosa for Emerging Adults (FT‐AN‐EA, age 18–25; Dodge et al. 2024) in a specialist outpatient Adult Eating Disorder Service. FT‐AN‐EA is adapted from adolescent FT‐AN (Eisler et al. 2016). Details of the emerging adult adaptations are presented in the method section below and are described in detail by Dodge et al. (2024).

Of note, FT‐AN and FBT, the model adapted in previous emerging adult studies, share many similarities (c.f., Gorrell et al. 2023 for comparison). Points of difference include the number of phases in each treatment (FT‐AN: 4 phases; FBT: 3 phases) and the additional emphasis in FT‐AN placed on engagement, neurobiology, formulation and preparing for endings (Baudinet et al. 2021a, 2021b). The emphasis in Phase 3 of FT‐AN on issues of both individual and family development could be considered to provide a broader focus on overall family functioning and dynamics that can be well adapted to the unique needs associated with emerging adulthood. Given that this aspect of treatment plays a central role in both short‐ and long‐term outcomes for this population group, it seems useful to present data on the outcomes for this adapted FT‐AN‐EA model.

## 2 Method

This study was approved by the South London and Maudsley (SLaM) Service Evaluation and Audit Committee (ref: 561). SLaM service evaluation and audit approval allows for analysis and publication of anonymised data extracted from case files without written consent from participants or carers. Outcome measures were administered as part of routine clinical care when possible. All methods were performed in accordance with the Declaration of Helsinki (World Medical Association Declaration of Helsinki 2013).

### 2.1 Design

This case series was conducted in a large specialist outpatient Adult Eating Disorder Service in London, UK. In 2021 this service increased its outpatient treatment offer to include FT‐AN. Potential patients from two referral pathways were prioritised: (1) those from the FREED pathway which accepts referrals from GPs and other healthcare providers for emerging adults, aged between 18 and 25 with a first episode of illness and an illness duration of less than 3 years; (2) patients with an age‐related service transition from child and adolescent services (CAEDS), due to turning 18. These CAEDS transition patients had all been receiving FT‐AN in their local specialist service and were given the option to continue with this. Most CAEDS transition patients choosing to continue with a family intervention were in the early phases of FT‐AN at the time of transition whilst two had been in treatment for more than 12 months.

Potential patients from the FREED pathway were identified at the time of referral and the option of FT‐AN explored at assessment. If they and their parent(s) were interested in FT‐AN they were provided with written information describing the treatment. As FT‐AN was a new intervention to be offered in the service, assessing clinicians were also provided with information through discussion and information sheets. Criteria for offering FT‐AN included: (1) a diagnosis of AN‐R, AN‐BP, or AAN; (2) living at home and agreeing to have family member(s) (usually parents) involved in both support at home and (3) able to attend therapy. For those who chose FT‐AN‐EA, treatment was offered either in‐person, online, or a combination of both. This allowed flexibility for families that would need to travel long distances to reach the clinic, as well as treatment being able to continue for those emerging adults who moved out of home during treatment (e.g., for work or study).

### 2.2 Participants

Patients who were assessed and completed treatment between April 2021 and December 2024 were recruited. All were assessed as appropriate for out‐patient treatment. Given the exploratory nature of this study and the newly established FT‐AN service, we cannot be sure that all potentially eligible referrals were systematically offered FT‐AN‐EA. Patients were selected through discussion between assessor, patient and family.

### 2.3 Intervention

The intervention offered, FT‐AN‐EA, is derived from FT‐AN (Eisler et al. 2016) and adapted for emerging adults. For full details of the treatment see Dodge et al. (2024). The main adaptations include:

— An overriding emphasis on collaboration between the EA, parents and clinician that places the EA at the centre of treatment.
— Opportunity for separated sessions for the EA and parents and greater flexibility with format throughout therapy to focus on individual and family functioning and development beyond specific eating disordered needs.
— Extended assessment including exploration with the EA of the pros and cons of parental involvement. Usually this would extend over two 50‐minute sessions.
— Providing a balance between increasing parental/carer support and the young person's wish for greater independence
— Exploring potential barriers to involvement on the part of parents and addressing any concerns
— Negotiating clarity around parent/carer involvement, respective roles and information sharing
— Appropriately involving siblings and/or partners in therapy, to support recovery and for exploration of needs related to family, couple and individual functioning.
— Addressing potential developmentally informed dilemmas around managing illness behaviours as the EA navigates this specific developmental stage (e.g., transition out of home, moving cities, university studies, romantic relationships, etc.).
— Preparing for endings that are appropriate to EA developmental stages. For example preparing recovery support plans that make use of appropriate levels of self‐monitoring, support from others and taking into account further plans such as; going to university, moving out of family home, returning to work, etc.

Treatment sessions were offered with one therapist for 50 minutes. Sessions initially commenced weekly and became less frequent as treatment progressed based on clinical improvement. This is different to the adapted FBT model described in other studies, with some offering 90 minute appointments (Dimitropoulos et al. 2018; Nyman‐Carlsson et al. 2020) and one delivering treatment sessions with two clinicians (Nyman‐Carlsson et al. 2020) who jointly provided 90 minute sessions.

Therapy was delivered by a small group of experienced clinicians (*n* = 7) from the FREED pathway, including family therapists, psychologists, and CBT therapists. Clinicians received FT‐AN training for adolescents and, thereafter, monthly supervision by a senior family therapist very experienced in FT‐AN and working with adults with eating disorders. There was also the option of live supervision for those less experienced in the model. This involved the possibility for the supervisor to join a session/sessions and offer live intervention and support of the lead therapist's work.

### 2.4 Analysis Plan

Participant data were extracted from clinical case notes. Variables extracted included baseline demographic information, referral source, BMI and treatment characteristics (e.g., treatment duration, number of sessions, completion rate). Paired sample *t*‐tests and Welch's test were used to assess change across treatment and between the FREED and CAEDS groups.

## 3 Results

A total of 109 patients with AN or AAN were referred to the FREED pathway between April 2021 and July 2023. Of these, thirty (27.5%) were referred for FT‐AN‐EA (21 initial FREED referrals, 9 transitions from CAEDS). From the 30 who started FT‐AN‐EA, two were classified as early non‐completers (withdrawn from FT‐AN‐EA after four sessions or fewer). One was referred to inpatient care due to significantly low weight (BMI < 15), the other was referred to the GP with a BMI > 18. Neither of their data are presented in Table 1 below, which shows treatment completers only.

**TABLE 1:** Demographic and treatment characteristics of treatment completers.

	FREED (*N* = 19)			CAEDS (*N* = 9)			Total (*N* = 28)		
	*N*	Mean	(SD, range)	*N*	Mean	(SD, range)	*N*	Mean	(SD, range)
Age									
Assessment	19	19.26	(1.66, 18–24)	9	18	(0.00, 18–18)	28	18.86	(1.48, 18–24)
BMI									
CAEDS assessment				9	16.36	(1.42, 14.2–19.3)			
Assessment	19	16.77	(1.72, 13.30–20.10)	9	18.34	(1.15, 16.3–19.9)	28	17.27	(1.71, 13.30–20.10)
Discharge	19	18.56	(2.14, 13.20–21.80)	9	18.89	(1.16, 17.4–21.1)	28	18.66	(1.86, 13.20–21.80)
Treatment characteristics									
Duration (months)	19	9.74	(4.21, 2–17)	9	11	(3.61, 6–19)	28	10.14	(4.01, 2–19)
Number of sessions	19	21.53	(7.62, 10–33)	9	22.67	(9.15, 8–34)	28	21.89	(7.99, 8–34)

	*N*	%	*N*	%	*N*	%
Gender						
Female	19	100	9	100	28	100
Male	0	0	0	0	0	0
DSM‐V diagnosis at assessment						
AN‐R	18	94.7	6	66.7	24	85.7
AN‐BP	1	5.3	1	11.1	2	7.1
A‐AN	0	0	2	22.2	2	7.1
Ethnicity						
White (British/European/Other)	13	68.4	8	88.9	21	75
Mixed/multiple ethnic groups	0	0	1	11.1	1	3.6
Asian/Asian British	4	21.1	0	0	4	14.3
Black/African/Caribbean/Black British	1	5.3	0	0	1	3.6
Other ethnic group	1	5.3	0	0	1	3.6
Discharged to						
GP	15	78.9	9	100	24	85.7
Other AEDS treatment	2	10.5	0	0	2	7.1
Inpatient	2	10.5	0	0	2	7.1

Abbreviations: A‐AN, atypical anorexia nervosa; AEDS, adult eating disorder service; AN‐BP, anorexia nervosa binge‐purge subtype; AN‐R, anorexia nervosa restrictive subtype; BMI, body mass index; CAEDS, child and adolescent eating disorder service; FREED, First Episode Rapid Early Intervention for Eating Disorders; GP, general practitioner.

Of the treatment completers (*n* = 28), BMI significantly increased from baseline to discharge with large effect size (*t*(27) = 4.59, *p* < 0.001, *d* = 0.87). At discharge, 20/28 patients (71.4%) restored weight to BMI > 18.5 kg/m^2^. Mean BMI change across treatment was 1.39 kg/m^2^ (SD = 1.60, range = −1.80 to 4.40) for all completers and 1.89 kg/m^2^ (SD = 1.57, range = −1.80 to 4.40) for those who started treatment underweight (*N* = 19, BMI < 18.5 kg/m^2^).

Treatment length ranged from 8 to 34 sessions over 2–19 months with a median of 22.5 sessions (IQR: 13.25–30) and 10 months (IQR: 8–13.25). Twenty‐four people (85.7%) were discharged to no further care (GP only) after FT‐AN‐EA, two (7.1%) were offered other interventions within the adult eating disorder service and two (7.1%) were referred to specialist inpatient treatment. One of those referred to inpatient care returned to outpatient FT‐AN and completed treatment thereafter, although their data are not included in this study.

At assessment, the CAEDS transition group were significantly younger (*t*(19.00) = 3.27, *p* = 0.004, *d* = 0.87) and had a significantly higher BMI (*t*(27) = 2.64, *p* = 0.014, *d* = 1.06). Mean treatment length with CAEDS prior to being seen in the adult service was 8 months (SD = 3.71) and mean BMI at assessment with CAEDS was 16.36 (SD = 1.42, range = 14.2–19.3). All CAEDS transition patients were discharged to GP care after completing FT‐AN‐EA with none receiving further intervention. There were no significant differences between the FREED and CAEDS transition group regarding treatment length (*t*(27) = 0.96, *p* = 0.35, *d* = 0.39) nor number of sessions (*t*(27) = 0.58, *p* = 0.57, *d* = 0.23) within the service, however CAEDS transition patients will likely have had previous treatment in adolescent services. See Table 1 for details.

## 4 Discussion

The study aimed to evaluate the applicability of adapted FT‐AN for EAs in a routine adult outpatient setting. The findings are encouraging and demonstrate most emerging adults gained weight during treatment. Not only was the amount of weight gained statistically significant, the majority (71.4%) were considered within the healthy weight range (BMI > 18.5 kg/m^2^) at discharge.

Most patients (85.7%) did not require more intervention and were discharged back to GP care, although two patients (7.1%) required inpatient treatment. Additionally, treatment non‐completion was very low (7.1%). As such, the current study provides preliminary data that conjoint working may be a useful model for emerging adults. Of note most of the cases included here were first episode recent onset cases, that is those with a good prognosis and many of the CAEDS transition patients started treatment at a higher weight compared to other FREED cohort studies (Austin et al. 2022; McClelland et al. 2018; Richards et al. 2023). Rates of weight‐recovery observed here (∼71% of total sample) were similar to those in FREED‐AN patients treated with individual therapies (CBT‐E or MANTRA; 52%–60%; McClelland et al. 2018; Austin et al. 2021; Richards et al. 2023). Similarly, the number of people in the current study requiring specialist inpatient treatment (∼7.1% inpatient) is comparable to FREED patients in previous studies requiring intensive day‐ or inpatient treatment (15.4%; Austin et al. 2022).

Notably, treatment length was quite variable. Some people needed relatively few sessions, whilst others attended more than 30. The median length of treatment (22 sessions) was slightly higher than that reported in child and adolescent studies (typical range 10–20 sessions over 6–12 months) (de la Poer Beresford et al. 2025) but briefer than in other studies of family‐based interventions in emerging adults (Chen et al. 2016; Dimitropoulos et al. 2018; Nyman‐Carlsson et al. 2020). It was also briefer than some adult individual treatments, such as CBT‐E, for people who are underweight (Dalle Grave and Pike 2023). Future research investigating the optimal treatment length would be of value, especially studies that can help unpick whether certain groups benefit from differing treatment lengths and the relationship of treatment length to outcomes.

Of particular interest is the group of people transitioning from CAEDS, given they had already had several months of intervention prior to attending the adult service and had a higher weight at baseline, yet had a similar duration of FT‐AN‐EA compared to other patients in this study. This could suggest that treatment almost needs to restart making age‐related transitions potentially more costly and wasteful of NHS resources reinforcing an argument for therapy to continue beyond the age of 18 within CAEDS where appropriate. The additional complexities faced by CAEDS transition patients that require a transition to adult services may require longer duration of therapy. Available qualitative indicate a strong therapeutic relationship and trust are important for treatment to be effective (Baudinet et al. 2024; James et al. 2025). The current findings suggest transitions, understandably, may interrupt this process and treatment may need to be longer to reestablish a good working relationship. Further research and discussion to understand the circumstances that necessitate transition could be undertaken.

Given the preliminary nature of this study and the non‐systematic recruitment strategy, findings need to be interpreted with caution. Many more emerging adults referred to the service during the recruitment period opted for an individual treatment, rather than FT‐AN‐EA. Unfortunately, data on what proportion were potentially eligible and then offered FT‐AN‐EA but declined are not available. Anecdotally, people declined FT‐AN‐EA due to not wanting family members involved in treatment and/or not wanting a similar type of treatment to what they had had in CAEDS. Also, most of the sample in the current study were at the lower end of the emerging adult range, suggesting that those at the upper end of the range may be less inclined towards FT‐AN‐EA and/or are more likely to be living independently. Additionally, clinician confidence in offering FT‐AN‐EA in an adult service where treatment is traditionally individual may be variable and may have influenced uptake.

### 4.1 Strengths and Limitations

This study adds preliminary data to the emerging literature supporting the efficacy of family‐focused interventions for emerging adults, thereby broadening the evidence‐based therapeutic options available for this population. In parallel, other studies have recently explored the utility of individual therapies such as CBT‐E and MANTRA in younger patients (below age 18) with AN (Dalle Grave and Calugi 2024; Wittek et al. 2023). Together, these initiatives will facilitate greater personalisation of treatment for adolescents and emerging adults with AN depending on developmental needs and other considerations (U. H. Schmidt et al. 2025). Nevertheless, the relatively small sample size, non‐systematic recruitment strategy, female only patient group, and reliance solely on weight data are major limitations. It was not possible to collect substantive patient reported outcome measure data, to present for discussion. Whilst there is strong consensus that recovery from AN includes both physical and psychosocial factors, weight recovery usually is a necessary pre‐condition for broader recovery. There was also large variability in the treatment length delivered. Future research should investigate FT‐AN‐EA further and compare its acceptability, efficacy and cost against evidence‐based individual therapies using a randomised controlled design. Furthermore, future studies could extend trials to young adults above the age of 25. In regards to CAEDS transition patients, data are limited regarding their BMI and treatment length prior to referral to adult services. Further research assessing the experiences of participants during CAEDS transitions to adult services could be beneficial. We were also unable to report on additional outcome measures, such as eating disorder psychopathology, depressive/anxiety symptoms, or family experiences.

### 4.2 Conclusions

FT‐AN‐EA is a promising intervention for emerging adults with anorexia nervosa both as new referrals to an adult service and at the time of transition from CAEDS. The current study provides preliminary data of its efficacy. More data are needed on the psychological impact of FT‐AN and its cost‐effectiveness in controlled trials.

## Funding

U. Schmidt receives salary support from the NIHR Mental Health Biomedical Research Centre (BRC) at the South London and Maudsley NHS Foundation Trust and King's College London (KCL). K. L. Allen and U. Schmidt are supported by the Medical Research Council/Arts and Humanities Research Council/Economic and Social Research Council Adolescence, Mental Health and the Developing Mind initiative as part of the EDIFY programme (Grant MR/W002418/1); and by the Medical Research Council Grant MR/X030539/1. J. Baudinet receives royalties from Routledge for a published adolescent treatment manual for multi‐family therapy for anorexia nervosa.

## Ethics Statement

Approval for this project was provided by the South London and Maudsley (SLaM) NHS Foundation Trust Service Evaluation and Audit Committee (ref: 561).

## Consent

Patients did not provide written consent, which is in line with UK service evaluation approval and covered under SLaM Service Evaluation and Audit approval.

## Conflicts of Interest

The authors declare no conflicts of interest.

## Permission to Reproduce Material From Other Sources

The authors have nothing to report.

## Data Availability

The data that support the findings of this study are available from the corresponding author upon reasonable request.
